# Dipeptidyl peptidase-4 inhibitor linagliptin attenuates neointima formation after vascular injury

**DOI:** 10.1186/s12933-014-0154-3

**Published:** 2014-11-19

**Authors:** Yuichi Terawaki, Takashi Nomiyama, Takako Kawanami, Yuriko Hamaguchi, Hiroyuki Takahashi, Tomoko Tanaka, Kunitaka Murase, Ryoko Nagaishi, Makito Tanabe, Toshihiko Yanase

**Affiliations:** Department of Endocrinology and Diabetes Mellitus, School of Medicine, Fukuoka University, 7-45-1 Nanakuma, Jonan-ku, Fukuoka 814-0180 Japan

**Keywords:** DPP-4 inhibitor, Linagliptin, Neointima formation, VSMC proliferation

## Abstract

**Background:**

Recently, glucagon-like peptide-1 (GLP-1)-based therapy, including dipeptidyl peptidase-4 (DPP-4) inhibitors and GLP-1 receptor agonists, has emerged as one of the most popular anti-diabetic therapies. Furthermore, GLP-1-based therapy has attracted increased attention not only for its glucose-lowering ability, but also for its potential as a tissue-protective therapy. In this study, we investigated the vascular-protective effect of the DPP-4 inhibitor, linagliptin, using vascular smooth muscle cells (VSMCs).

**Methods:**

Six-week-old male C57BL/6 mice were divided into control (n =19) and linagliptin (3 mg/kg/day, n =20) treated groups. Endothelial denudation injuries were induced in the femoral artery at 8 weeks of age, followed by evaluation of neointima formation at 12 weeks. To evaluate cell proliferation of rat aortic smooth muscle cells, a bromodeoxyuridine (BrdU) incorporation assay was performed.

**Results:**

Linagliptin treatment reduced vascular injury-induced neointima formation, compared with controls (*p* <0.05). In these non-diabetic mice, the body weight and blood glucose levels did not change after treatment with linagliptin. Linagliptin caused an approximately 1.5-fold increase in serum active GLP-1 concentration, compared with controls. In addition, the vascular injury-induced increase in the oxidative stress marker, urinary 8-OHdG, was attenuated by linagliptin treatment, though this attenuation was not statistically significant (*p* =0.064). Moreover, linagliptin did not change the serum stromal cell-derived factor-1α (SDF-1α) or the serum platelet-derived growth factor (PDGF) concentration. However, linagliptin significantly reduced *in vitro* VSMC proliferation.

**Conclusion:**

Linagliptin attenuates neointima formation after vascular injury and VSMC proliferation beyond the glucose-lowering effect.

## Background

Patients with diabetes mellitus have a greater risk of cardiovascular events compared with non-diabetic subjects [1], and they frequently experience restenosis after coronary angioplasty, even if intervention is performed with currently established drug-eluting stents [2]. Consequently, the aim of glycemic control is not only lowering the blood glucose level, but also improving quality of life and mortality by preventing the occurrence and progression of vascular complications. Thus, it is important to investigate the vascular-protective effect of anti-diabetic agents.

Incretins, glucagon-like peptide-1 (GLP-1) and glucose-dependent insulinotropic polypeptide (GIP) act on pancreatic β cells to stimulate glucose-responsive insulin secretion, but also have tissue-protective effects beyond lowering blood glucose levels [3], such as cardiovascular protection [4], anti-hepatic steatosis [5] and anti-Alzheimer’s disease [6].

GLP-1-based therapy, which includes dipeptidyl peptidase-4 (DPP-4) inhibitors and GLP-1 receptor agonists, has become a popular treatment for patients with type 2 diabetes. Indeed, the DPP-4 inhibitor is one of the most prescribed anti-diabetic agents in Japan, because of its efficacy and safety [7]. Pooled analysis of clinical studies revealed that sitagliptin has a good tolerability in elderly patients with type 2 diabetes [8], with a lower rate of cardiovascular-related events compared with sulphonylurea [9]. Linagliptin is a newly identified, biliary excreted DPP-4 inhibitor, which has recently been approved as a once-daily oral glucose-lowering agent [10]. Similar to sitagliptin, the cardiovascular safety of linagliptin has been suggested to be acceptable in high-risk patients [11], and its efficacy and safety are acceptable as an add-on therapy to metformin and sulphonylurea [12]. Interestingly, in retrospective analysis, linagliptin reduced cardiovascular events compared with other glucose-lowering agents [13]. Several experiments have revealed that the vascular-protective effect of linagliptin involves incretin-dependent and -independent mechanisms, such as anti-oxidative stress [14], inhibition of advanced glycation end products (AGE) and the receptor for AGE axis [15], and inhibition of vascular DPP-4 activity [16]. On the other hand, we have previously reported the vascular-protective effects of exendin-4, a GLP-1R agonist, including attenuation of atheroma formation in apoE-deficient mice via inhibition of NFκB activation in macrophages [17], and reduction of neointima formation after vascular injury via 5′ AMP-activated protein kinase activation in VSMCs [18]. Furthermore, we have recently demonstrated the anti-cancer effect of exendin-4 using a prostate cancer model [19]. However, recent clinical studies have unfortunately failed to prove cardiovascular benefits of DPP-4 inhibitors in patients with prior cardiovascular events using saxagliptin [20] or alogliptin [21]. These data prompted us to examine whether the DPP-4 inhibitor, linagliptin, can attenuate neointima formation after vascular injury, which is one of the experimental models for coronary restenosis after coronary angioplasty. Furthermore, currently there are no reports on whether linagliptin can attenuate neointima formation and VSMC proliferation after vascular injury. Thus, we examined the vascular-protective effect of linagliptin, using a VSMC proliferation model both *in vivo* and *in vitro*.

## Methods

### Animals

The study protocol was reviewed and approved by the Animal Care and Use Committee of Fukuoka University. Six-week-old male C57BL/6 mice were purchased from Oriental Yeast (Tokyo, Japan). All mice were housed in a polycarbonate cage with a wooden chip mat on the floor, and water was available *ad libitum*. C57BL/6 mice were divided into two groups, control (n =19) and linagliptin treatment (n =20), which was kindly provided by Boehringer Ingelheim Pharma GmbH & Co. KG (Biberach an der Riss, Germany). At 6 weeks of age, control mice were fed normal chow (22.6% protein, 53.8% carbohydrate, 5.6% fat, 6.6% mineral and vitamin mixture, and 3.3% fiber; in total: 356 kcal/100 g) with vehicle, and linagliptin-treated mice were fed normal chow with linagliptin (0.083 g/kg chow, which results in a mean plasma level of 50–150 nM, corresponding to an oral dose of 3 mg/kg/day) for 6 weeks. The animal room was kept on a 12-h light/dark cycle at a constant temperature (22 ± 1°C) and relative humidity of 55 ± 5% throughout the experimental period. Endothelial denudation injuries were induced in the femoral artery at 8 weeks of age, followed by evaluation of neointima formation at 12 weeks of age.

### Guidewire-induced endothelial denudation injury

Mouse femoral artery endothelial denudation injury was induced in C57BL/6 mice of the control and linagliptin groups at 8 weeks of age, as previously described [18,22]. Briefly, endovascular injury was induced by four passages of a 0.25-mm SilverSpeed-10 hydrophilic guidewire (Micro Therapeutics Inc., Irvine, CA, USA) into the left femoral artery. Sham surgery without injury was performed on the contralateral right side. Mice were euthanized 4 weeks after injury, and femoral arteries were isolated for tissue analysis.

### Tissue preparation and morphometry

Following sacrifice, mice were perfused via a cannula in the left ventricle with phosphate-buffered saline for 5 min, followed by 4% paraformaldehyde for 30 min at 100 cm H_2_O. The femoral arteries were embedded in paraffin and cut into 5-μm sections for further analysis. Serial sections of the 1.5-mm proximal region from the incision site of the wire insertion and the sham surgery vessels on the other side were evaluated using an Elastica van Gieson stain kit (4033–4037, Muto Pure Chemicals Co., Tokyo, Japan), to visualize the internal elastic lamina, as previously described [18]. Specimens were viewed under a microscope (BZ9000; Keyence, Tokyo, Japan) connected to a computer. The intimal and medial areas were measured by computerized morphometry using the software BZ-II analyzer (Keyence, Tokyo, Japan). Intimal hyperplasia was defined as the formation of a neointimal layer medial to the internal elastic lamina. The medial area represents the area between the external elastic lamina and the internal elastic lamina. The intima-to-media ratio was calculated as the intimal area divided by the media area, as described previously [18,22].

### Intraperitoneal glucose tolerance test (IPGTT)

IPGTTs were performed on C57BL/6 mice with or without linagliptin treatment after a 12-h fast. Glucose (2 g/kg) was administered intraperitoneally. Plasma glucose level was measured using the glucose oxidase method by a compact glucose analyzer (Glutest Neo Super, Sanwa Chemical Co., Kanagawa, Japan).

### Laboratory data

Blood samples were collected at euthanasia. Serum active GLP-1 concentration was measured using an insulin enzyme-linked immunosorbent assay (ELISA) kit (IBL, Osaka, Japan). In addition, the serum stromal cell-derived factor-1α (SDF-1α) and platelet-derived growth factor (PDGF) concentration was measured using ELISA kits DSA00 and MBB00, respectively (R&D Systems, Minneapolis, MN, USA). Urinary 8-hydroxy-2’-deoxyguanosine (8-OHdG) concentration was measured using a kit (JaICA, Shizuoka, Japan). Urinary creatinine levels were also measured using a kit (Wako, Osaka, Japan) to adjust the urine concentration, as previously described [23].

### BrdU assay

To evaluate cell proliferation of rat aortic smooth muscle cells, the bromodeoxyuridine (BrdU) incorporation assay was performed using a Cell Proliferation ELISA kit (1647229; Roche Applied Science, Penzberg, Germany), as previously described [18,19]. Briefly, rat aortic smooth muscle cells were plated at 2000 cells/well in 96-well culture plates in complete media (n =5), and were incubated in Dulbecco’s Modified Eagle Medium (DMEM) with 10% fetal bovine serum (FBS). After attaining 60–70% confluence, rat aortic smooth muscle cells were incubated in DMEM containing 0.1% FBS with or without 10 nM linagliptin for 48 h, and then stimulated with PDGF (25 ng/ml; Sigma-Aldrich, St Louis, MO, USA) or DPP-4 (200 ng/ml; R&D Systems) for 24 h. BrdU solution (10 μM) was added during the last 2 h of stimulation. Next, the cells were dried and fixed, and cellular DNA was denatured with FixDenat solution (Roche Applied Science) for 30 min at room temperature. A peroxidase-conjugated, mouse anti-BrdU monoclonal antibody (Roche Applied Science) was added to the culture plates and incubated for 90 min at room temperature. Finally, tetramethylbenzidine substrate was added for 15 min at room temperature and absorbance of the samples was measured using a microplate reader at 450–620 nm. Mean data are expressed as a ratio of control (non-treated) cell proliferation.

### Statistical analysis

Unpaired *t-*tests were performed for statistical analysis as appropriate. *p* values lower than 0.05 were considered to be statistically significant. Results are expressed as mean ± SEM.

## Results

### Linagliptin attenuates neointima formation after vascular injury

**Figure 1:**
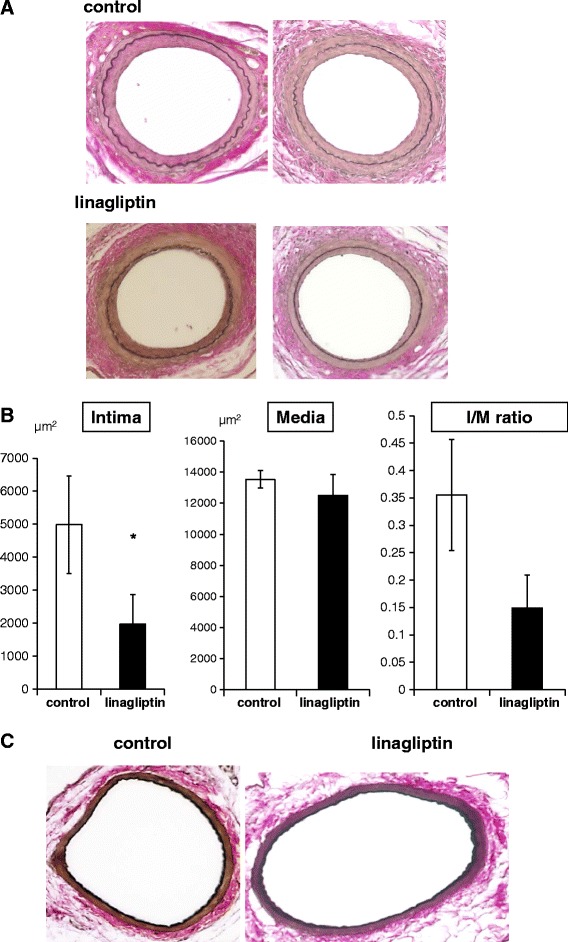
**Neointima formation after vascular injury in control and linagliptin-treated mice.** Endothelial denudation injuries were induced in the left femoral artery of control (n =19) and linagliptin-treated mice (n =20). **(A)** Tissues were evaluated by staining with Elastica van Gieson, to visualize the internal elastic lamina (magnification, ×200). **(B)** The area of intima, media and intima/media was calculated for each group. Data are mean ± SEM. *p <0.05 vs. control. **(C)** Sham control vessels were stained with Elastica van Gieson.

C57BL/6 mice were treated with control or linagliptin (3 mg/kg/day) from 6 weeks of age to 12 weeks of age. Mouse femoral artery endothelial denudation injuries were performed at 8 weeks of age in both groups, and then neointima formation was evaluated at 12 weeks of age. Endothelial denudation injury in the control mice resulted in considerable neointima formation. In contrast, neointima formation was substantially reduced by linagliptin treatment (Figure 1A). Specifically, as shown in Figure 1B, quantitative analysis revealed a 60.6% reduction in neointima formation in linagliptin-treated mice compared with that in control mice, (control: 4989 ± 1478 vs. linagliptin: 1967 ± 894 μm^2^, *p* <0.05), although the media layer areas were not different (control: 13534 ± 568 vs. linagliptin: 12500 ± 1355 μm^2^). The intima/media (I/M) ratio was lower in the linagliptin-treated mice compared with that in the control mice, but the difference was not statistically significant (control: 0.36 ± 0.10 vs. linagliptin: 0.15 ± 0.06, *p* =0.059; Figure 1B). In addition, right vessels with sham surgery did not change by linagliptin treatment (Figure 1C). These data suggest that linagliptin attenuates vascular injury-induced neointima formation *in vivo* in non-diabetic mice.

### Linagliptin does not change body weight and blood glucose level in non-diabetic mice

**Figure 2:**
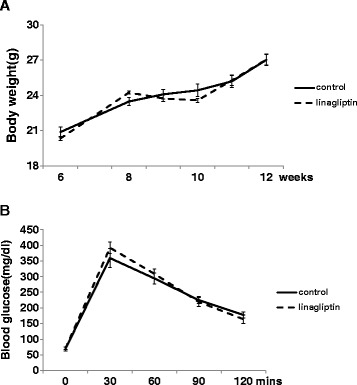
**Body weight and IPGTT in control and linagliptin-treated mice.** Comparison of the change in body weight **(A)** and IPGTT **(B)** in the control group (n =14) and the linagliptin-treated group (n =14). Data are mean ± SEM.

To investigate whether linagliptin changes body weight and blood glucose in non-diabetic mice, we measured mice body weight and performed IPGTT. As shown in Figure 2A and B, there were no differences between the control mice and the linagliptin-treated mice in body weight or glucose tolerance, suggesting that linagliptin attenuated neointima formation independent of the glucose-lowering effect and body weight reduction.

### Linagliptin increases serum active GLP-1 concentration, but not SDF-1α and PDGF

**Figure 3:**
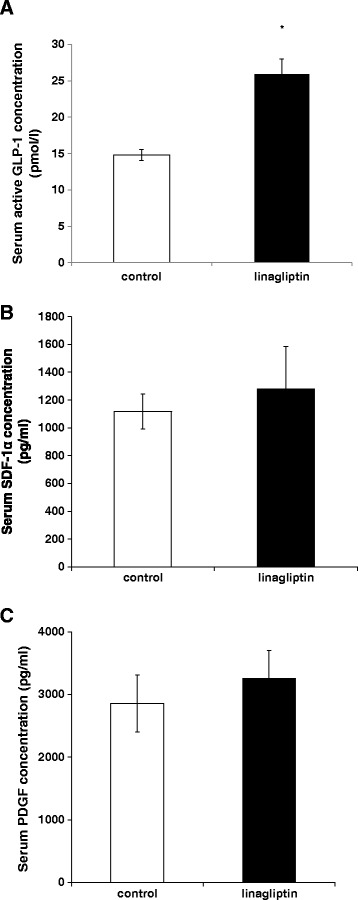
**Serum active GLP-1, SDF-1α and PDGF concentration in control and linagliptin-treated mice. (A)** Serum active GLP-1 concentration in control (n =14) and linagliptin-treated mice (n =14), **(B)** serum SDF-1α concentration in control (n =9) and linagliptin-treated mice (n =10), and **(C)** serum PDGF concentration in control (n =9) and linagliptin-treated mice (n =8), assessed using ELISA kits. Data are mean ± SEM. *p <0.05 vs. control.

To examine whether orally administered linagliptin can act as a DPP-4 inhibitor to increase active GLP-1 in non-diabetic mice, we measured the serum active GLP-1 concentration in linagliptin-treated and control mice. As shown in Figure 3A, the serum active GLP-1 concentration increased by 1.5-fold in the linagliptin-treated group compared with that in the control group (control: 14.79 ± 0.72 pmol/L vs. linagliptin: 25.97 ± 1.99 pmol/L, *p* <0.001). In addition to its effect on GLP-1, DPP-4 inhibitors also confer vascular protection by inducing DPP-4 substrates [24]. SDF-1α, which is one of the DPP-4 substrates that is degraded by DPP-4 through its cleavage [25], is a chemokine inducing endothelial progenitor cells to differentiate into endothelial cells to protect the vasculature, and increased SDF-1α concentration has been reported to be a mechanism by which DPP-4 inhibitors protect the cardiovascular system [26]. Hence, we measured the serum SDF-1α concentration, as described previously [27]. However, linagliptin did not increase the serum SDF-1α level in mice (Figure 3B). Furthermore, the serum PDGF concentration, the most powerful proliferator signal for VSMCs, was also unaltered by linagliptin treatment (Figure 3C). These data suggest that linagliptin increases the serum active GLP-1 concentration, but may not change other serum factors related to VSMC proliferation, including DPP-4 substrates.

### Linagliptin decreases urinary 8-OHdG concentration after vascular injury

**Figure 4:**
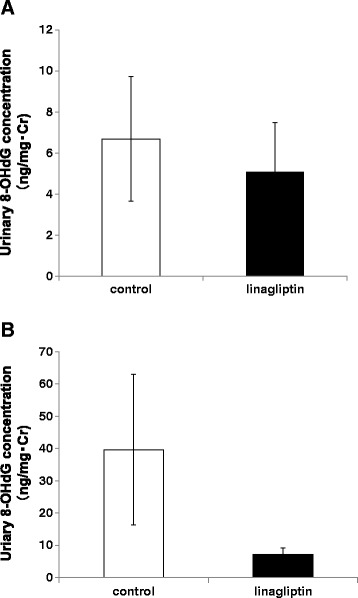
**Urinary 8-OHdG concentration in the control and linagliptin groups before and after vascular injury.** Comparison of urinary 8-OHdG concentration in the control (n =9) and linagliptin-treated mice (n =8) **(A)** before and **(B)** after vascular injury using an ELISA kit. Urinary 8-OHdG concentration was adjusted using urinary creatine. Data are mean ± SEM.

Oxidative stress is one of the most important accelerators of VSMC proliferation [28], and a previous report has suggested that linagliptin has anti-oxidative effects independent of the glucose-lowering effect [14]. Thus, we examined the oxidative stress marker, urinary 8-OHdG, before and after vascular injury with or without linagliptin treatment. As shown in Figure 4A, the urinary 8-OHdG level was not different between the control and linagliptin-treated mice before vascular injury (control: 6.68 ± 3.03 ng/mgCr vs. linagliptin: 5.08 ± 2.41 ng/mgCr). In contrast, the urinary 8-OHdG level after vascular injury decreased by linagliptin treatment (control: 39.61 ± 23.37 ng/mgCr vs. linagliptin: 7.18 ± 1.95 ng/mgCr; Figure 4B), but not significantly (*p* =0.064). These data suggest that anti-oxidative stress effects may be a potential mechanism by which linagliptin attenuates neointima formation after vascular injury.

### Linagliptin reduces VSMC proliferation *in vitro*

**Figure 5:**
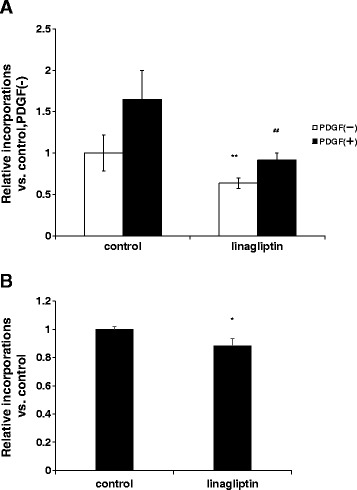
**BrdU assay using rat aortic smooth muscle cells. (A)** After rat aortic smooth muscle cells were plated, the cells were incubated in DMEM containing 0.1% FBS with or without 10 nM linagliptin for 48 h. Subsequently, cells were stimulated with PDGF (25 ng/ml) for 24 h. **(B)** Cells were incubated in DMEM with 0.1% FBS for 24 h. Subsequently, they were stimulated with DPP-4 (50 ng/ml) with or without 10 nM linagliptin for 24 h. BrdU solution was added during the last 2 h, and cells were harvested for measurement of DNA synthesis using a microplate reader at 450–620 nm. Mean data are expressed as the ratio of the control cell proliferation. Unpaired *t*-tests were performed to calculate statistical significance. Data are mean ± SEM. **p <0.01 vs. control PDGF(−), ^##^ *p* <0.01 vs. control PDGF(+), *p <0.05 vs. control.

To investigate the direct effect of linagliptin, we performed BrdU incorporation assays on rat aortic smooth muscle cells. As shown in Figure 5, linagliptin reduced BrdU incorporation in rat aortic smooth muscle cells both with and without PDGF stimulation compared with that in the controls, suggesting that linagliptin reduces VSMC proliferation in both the quiescent and growth factor-stimulated states. Because a recent report has suggested that DPP-4 stimulates VSMC proliferation directly [29], we examined whether linagliptin attenuates DPP-4-induced VSMC proliferation. As shown in Figure 5B, linagliptin attenuated DPP-4-induced VSMC proliferation.

## Discussion

The present study demonstrated that the DPP-4 inhibitor, linagliptin, increased the serum active GLP-1 concentration and attenuated neointima formation after vascular injury independent of its glucose-lowering effect. Our experiments suggest the mechanisms of action include an anti-oxidative stress effect and a direct inhibitory effect of linagliptin on VSMC proliferation.

Because the aim of glycemic control is not only reduction of blood glucose level or hemoglobin A1c, but also prevention of vascular complications, it is very important to elucidate the pleiotropic or vascular-protective effect of anti-diabetic agents. GLP-1-based therapy has attracted increased attention, because of its tissue-protective effect beyond glycemic control [3-6]. Inhibition of neointima formation after vascular injury by the DPP-4 inhibitor, sitagliptin, has been reported in a rat cervical artery balloon injury model [30]. In addition, we have previously reported that the DPP-4 inhibitor, anagliptin, attenuated VSMC proliferation in non-diabetic apoE-deficient mice [27]. Thus, inhibition of VSMC proliferation may be one important effect of DPP-4 inhibitors. Although we have previously observed a similar anti-neointima formation effect with the GLP-1 receptor agonist, exendin-4, in the present study we observed other mechanisms by which linagliptin could induce vascular-protective effects independent on GLP-1. Firstly, linagliptin attenuated VSMC proliferation directly with or without growth factor and DPP-4-induced VSMC proliferation (Figure 5). Lamers *et al*. have reported that DPP-4 is released as an adipokine from obese visceral fat, and stimulates VSMC proliferation through extracellular signal-regulated kinase (ERK)/mitogen-activated protein kinase (MAPK) phosphorylation in VSMCs [29]. According to this report and our present data, DPP-4 inhibitors may attenuate VSMC proliferation by directly inhibiting DPP-4 independent of incretins. Secondly, linagliptin reduced oxidative stress in mice after vascular injury (Figure 4B). Linagliptin is a unique biliary excreted DPP-4 inhibitor, which has anti-oxidative stress effects because of its xanthine structure [10], long half-life and widespread tissue distribution [31]. The anti-oxidative stress effect and associated vascular protection of linagliptin is an important characteristic, because diabetic patients often have high oxidative stress that can result in atherosclerosis [32]. As we have previously reported, diabetic patients have a higher mitochondrial DNA somatic mutation ratio [33], a marker of long-term oxidative stress, which is also associated with atherosclerosis [34]. In addition, we have previously shown that oxidative stress induces further insulin resistance through tyrosine nitration of insulin receptor substrate-1, resulting in reduced glucose disposal [35]. These data suggest that oxidative stress is a key trigger of the vicious cycle between insulin resistance, hyperglycemia and vascular complications. In the present study, we observed a reduction in urinary 8-OHdG after vascular injury with linagliptin treatment, though it was not statistically significant, probably because we used non-diabetic and non-septic, normal mice.

In addition, DPP-4 cleaves not only incretins, but also other substrates such as SDF-1α, peptide YY, and brain natriuretic peptide (BNP) [24]. Of these substrates, SDF-1α is the most potent chemokine known to induce cardiovascular protection by DPP-4 inhibitors, because it has been reported that the levels of serum SDF-1α and endothelial progenitor cells increased with oral DPP-4 inhibitor treatment in patients with type 2 diabetes [36]. Accordingly, we hypothesized that linagliptin attenuates neointima formation after vascular injury through induction of endothelial progenitor cells by increasing SDF-1α. However, linagliptin did not increase the serum SDF-1α levels. This is consistent with our previous report showing that anagliptin did not increase the serum SDF-1α levels in mice [27]. The difference in the results between the studies mentioned above is likely because of the different species investigated, human vs. rodent. In the present study, we could not measure other DPP-4 substrates, such as BNP [24], because of sample limitation. Further elucidation of DPP-4 substrates is required. Furthermore, Kanasaki *et al.* have recently reported that linagliptin inhibited the endothelial-to-mesenchymal transition in type 1 diabetic mouse kidney, independent of the glucose-lowering effect [37]. This pleiotropic effect could be a potential effect of linagliptin. In the present study, we were unable to investigate all mechanisms by which linagliptin attenuates neointima formation after vascular injury. Further studies are needed to clarify the vascular-protective effect of linagliptin.

## Conclusion

The DPP-4 inhibitor, linagliptin, attenuates neointima formation after vascular injury independent of its glucose-lowering effect, possibly through anti-oxidative stress effects, increasing the serum active GLP-1 concentration, attenuating DPP-4-induced VSMC proliferation and direct inhibition of VSMC proliferation.

## Abbreviations

8OHdG: 8-hydroxy-2’-deoxyguanosine
AGE: Advanced glycation end products
BrdU: Bromodeoxyuridine
DPP-4: Dipeptidyl peptidase 4
GIP: Glucose-dependent insulinotropic polypeptide
GLP-1: Glucagon-like peptide-1
IPGTT: Intraperitoneal glucose tolerance test
PDGF: Platelet-derived growth factor
SDF-1α: Stromal cell-derived factor-1 α
VSMC: Vascular smooth muscle cells
